# The Quality Evaluation of Highland Barley and Its Suitability for Chinese Traditional Tsampa Processing

**DOI:** 10.3390/foods13040613

**Published:** 2024-02-18

**Authors:** Hu Xia, Bo Yu, Yanting Yang, Yan Wan, Liang Zou, Lianxin Peng, Lidan Lu, Yuanhang Ren

**Affiliations:** Key Laboratory of Coarse Cereal Processing, Ministry of Agriculture and Rural Affairs, Sichuan Engineering and Technology Research Center of Coarse Cereal Industralization, College of Food and Biological Engineering, Chengdu University, Chengdu 610106, China

**Keywords:** highland barley, physicochemical traits, Tsampa, quality evaluation, predictive model

## Abstract

The physicochemical traits of highland barley prominently affect the quality of Tsampa. To find out the relevance between the physicochemical properties of raw material and the texture parameters of processed products, twenty-five physicochemical traits and ten quality parameters for seventy-six varieties of highland barley were measured and analyzed. The results showed that there was a significant difference between the physicochemical indexes for highland barleys of various colors. The dark highland barley generally has more fat, protein, total dietary fiber, phenolic, Mg, K, Ca, and Zn and less amylose, Fe, Cu, and Mo than light colored barley. Then, these highland barleys were made into Tsampa. A comprehensive quality evaluation model based on the color and texture parameters of Tsampa was established through principal component analysis. Then, cluster analysis was used to classify the tested samples into three edible quality grades predicated on the above evaluation model. At last, the regression analysis was applied to establish a Tsampa quality predictive model according to the physicochemical traits of the raw material. The results showed that amylose, protein, β-Glucan, and a* and b* could be used to predict the comprehensive quality of Tsampa. The predicted results indicated that 11 of 14 validated samples were consistent with the actual quality, and the accuracy was above 78.57%. Our study built the approach of the appropriate processing varieties evaluation. It may provide reference for processing specific highland barley.

## 1. Introduction

Highland barley (*Hordeum vulgare* L. var. *nudum* Hook. f., HB) is a variety of barley, called “Qingke” in Chinese and “ནས། (Ne)” in Tibetan [1]. Since the seeds and glumes separate at harvest, it is also known as hull-less barley or naked barley. Because of the characteristics of strong cold resistance, short growth period, high yield, early development, and wide adaptability, HB is one of the few crops that can grow in the Qinghai–Tibet Plateau [2]. Hence, HB is an important and traditional staple food for Tibetans [3]. HB contains wide-ranging nutrients, such as bioactive carbohydrates and polyphenols, minerals, vitamins, phenolic, flavonoids, and β-glucan. The unique composition of HB contributes to its various health benefits, such as anti-inflammation, anticancer, antidiabetic, antibacterial, antiobesity, antifatigue, antiaging, hyperglycaemia, and hyperlipidemia [4]. Therefore, HB has attracted widespread attention due to the increasing emphasis on health, and its processed products are becoming increasingly popular in recent years.

Tsampa, also known as “Zanba” in Chinese, is a traditional staple food for people in the Qinghai–Tibet Plateau and it is also the main processed products of HB. Tsampa is easy to cook and carry. It can provide both sufficient nutrition and energy meaning it has become the most practical food form in the specific environment of the alpine zone [1]. Previous research showed that the high-quality processed products were closely related to physicochemical properties of raw materials [5]. Taking soybeans and tofu as an example, total protein, glycinin (11S), glutamate, water-soluble protein, α-subunit, linoleic acid, and tyrosine contents were key factors affecting tofu quality. Glycinin (11S) and β-conglycinin (7S) both contribute differently to the functional characteristics of soybean protein, but the ratio of 11S to 7S influences the tofu’s quality. Moreover, it has been reported that there was a negative correlation between the oil content in soybeans and the hardness of the tofu.

Therefore, the quality of Tsampa is also affected by multiplex HB varieties due to its different physicochemical properties which are interrelated to its processing characteristics [2]. For instance, previous studies have shown that there are significant differences in the starch composition of different HB varieties, which exhibit different properties during processing [2]. Dough made from high amylose starch has higher water absorption compared to traditional wheat flour [6], with weak stability and gluten strength [7,8]. Therefore, building the relationship between Tsampa quality and the HB physicochemical properties is necessary, and it would be beneficial to selecting suitable HB varieties for specific processing products.

In our study, 25 physicochemical traits of raw material and 10 texture parameters of Tsampa for different HB varieties were systematically evaluated. Then, principal component analysis and cluster analysis were used to establish the Tsampa quality evaluation model and classify Tsampa into three grades. Finally, regression analysis was used to create a prediction model linking the Tsampa comprehensive quality and HB physicochemical properties. This study determines the approach for the evaluation of HB-appropriate processing and may be conducive to processing specific HB.

## 2. Materials and Methods

### 2.1. Materials

The 76 varieties of HB were collected and purchased from Tibet, Qinghai, Sichuan, and Yunnan of China. For detailed information on the tested HB, refer to Table S1. The HB powder was obtained from the HB whole seeds, pulverized with a multifunctional grinder (BJ-800A, Deqing Baijie Electrical Appliances Co., Ltd., Zhejiang, China), passed through a 65-mesh sieve, and stored at 4 °C.

### 2.2. Seeds Color

A high-quality colorimeter (NH310, 3NH, Guangdong, China) was used to determine the lightness (L*), redness (a*), and yellowness (b*) of HB seeds. We randomly took readings at five positions after spreading the sample over the entire container, and the values were averaged. L* indicates brightness, and its value ranges from 0 (pure black) to 100 (pure white). Next, a* and b* represent the red-green and blue-yellow scales, respectively; the value is less than 0 for green or blue, and greater than 0 for red or yellow [9]. Each value is the average of five random positions’ readings of the sample.

### 2.3. Moisture Content

The moisture content was determined by air oven method [10] Briefly, we placed 5.00 g of each sample into the drying oven at 103–104 °C until the sample weight was unchanged. The loss of weight was used to calculate the moisture content [11].

### 2.4. Fat Content

For the determination of the fat content, we referred to the method used by B. Uzun [12], with slight modifications. Each sample (2 g powder) was subjected to oil extraction by Soxhelt apparatus (SZT-06A, Suzhou Tianwei Instrument Co., Ltd., Suzhou, China). The fat content was determined by the difference in weight of the samples before and after treatment.

### 2.5. Starch Composition

The total starch content was determined using a starch content assay kit (BC0705, Solarbio, Beijing, China) based on anthrone sulfate chromogenic method [13]. Briefly, 80% ethanol was used to separate the soluble sugar and starch in the sample. Then, the starch was converted into glucose by acid hydrolysis. The starch content was calculated by measuring the glucose content by anthrone colorimetry.

The amylose content was determined using an amylose content assay kit (BC4265, Solarbio, Beijing, China) based on iodine colorimetry [14]. Briefly, the sample was dispersed in ethanol to remove soluble sugars. Then, it continued to be washed by diethyl ether to remove fat. The amylose content determination was performed using iodine colorimetry after samples were gelatinized with sodium hydroxide.

By deducting the amylose content from the total starch content, the amylopectin content was determined.

### 2.6. Protein Content

The protein content was determined using a plant protein extraction kit (BC3720, Solarbio, Beijing, China) and BCA protein assay kit (PC0020, Solarbio, Beijing, China). The HB seeds were crushed with liquid nitrogen. Then, the cracking agent involved protease inhibitors and PMSF for protein extraction. The obtained protein was quantified using a bicinchoninic acid (BCA) assay.

### 2.7. β-Glucan Content

Total β-Glucan content was determined with the mixed-linkage (1–3) (1–4) β-D-glucan assay kit (Megazyme International, Bray, Ireland) following the streamline [10].

### 2.8. Total Dietary Fiber Content

Total dietary fiber content was determined using the total dietary fiber enzymatic–gravimetric method [10]. Briefly, the dried sample was enzymolyzed by thermostable α-amylase, protease, and amyl glucosidase. The sample was precipitated using ethanol and then filtered. The residue was washed with 78% ethanol, 95% ethanol, and acetone in turn. The total dietary fiber content was calculated by subtracting the weight of protein and ash from the filtered and dried residue.

### 2.9. Extraction of Free and Bound Phenolic

Extraction of free phenolics was based on the reported method with slight modifications [15]. Briefly, we placed 0.1 g of the sample into 2.5 mL of 80% chilled acetone, and extracted the sample with an ultrasonic machine (KQ-400E, Kun Shan Ultrasound Instrument Co., Suzhou, China) at 500 W for 20 min. Then, we centrifuged the sample at 4000 r/min for 10 min with a high-speed centrifuge (SF-TGL-16W, Feiqiaer Analytical Instruments Co., Ltd., Shanghai, China). We collected the supernatant and repeated the extraction process twice, fixed the extract to 10 mL, and stored it at 4 °C to protect it from the light.

Extraction of bound phenolics was based on the reported method with slight modifications [16]. We digested the residue from the free phenolic extract with 1.5 ml 2 mol/L sodium hydroxide at room temperature for 1 h away from light. Next, 2 mol/L of hydrochloric acid was used to neutralize the pH of the mixture. Then, ethyl acetate was used to extract, and the process was repeated five times. We collected the ethyl acetate layer and removed the solvent with nitrogen blowing. We redissolved the residue to 10 mL with chilled acetone and stored it at 4 °C away from the light.

### 2.10. Phenolic Content

The contents of the free and bounded phenolics were determined with a plant total phenol content assay kit (BC1340, Solarbio, Beijing, China) based on the Folin–Ciocalteu method. The content value is on behalf of gallic acid (mg GAE/100 g). The total phenolic content was calculated as the sum of the free and bounded phenolic.

### 2.11. Element Analysis

Element analysis was based on the reported method with slight modifications [17]. We transferred 0.1 g of HB powder into a digestion tube with 10 mL of concentrated nitric acid (Trace Metal Grade, Merck, Darmstadt, Germany). The tube was placed on a graphite digestion apparatus (DS32-260, CIF Instruments Chengde Co., Ltd., Chengde, China). After digestion at 120 °C for 1 h, we raised the temperature to 150 °C and kept going for 2 h. Then, we kept the temperature at 200 °C until the solution was almost dry. Using 1% dilute nitric acid, we diluted the sample to 50 mL before detection. The element (Na, Mg, K, Ca, Mn, Fe, Co, Cu, Zn, Se, Mo) content was analysed by inductively coupled plasma-mass spectrometry (iCAP RQ, Thermo Fisher, Waltham, MA, USA) according to the reported method [18].

### 2.12. Preparation of Tsampa

Due to the scarcity of some samples, only 70 parts of HB were processed to Tsampa, continuously. We rinsed 100 g of HB seeds at room temperature and air dried them. Then the seeds were stir-fried at 220–260 °C until the fissure ratio of seeds exceeded 85% or seeds stopped expanding. After cooling at room temperature, the stir-fried seeds were pulverized and passed through a 65-mesh sieve. This flour was mixed with sugar, ghee, and milk at a ratio of 10:4:1:10 and was then kneadedinto a dough. Finally, using a model, we shaped the dough into a cylinder (r = 1 cm, h = 1 cm).

### 2.13. Color of Tsampa

The color of Tsampa was determined by the colorimeter; refer to the method in Section 2.2.

### 2.14. Texture Parameters of Tsampa

The texture parameters (hardness, adhesiveness, springiness, cohesiveness, gumminess, chewiness, and resilience) of Tsampa were evaluated by the texture analyzer (TA-XTplusC, Stable Micro Systems Ltd., Godalming, Surrey, UK) with a P36 aluminum cylindrical probe. For the test methods and instrument parameters, refer to the previous report [19].

### 2.15. Statistical Analysis

Apart from the dietary fiber content test, which was set at one measurement, all other experiments were repeated at least three times with a completely randomized design. One-way ANOVA was used to determine the significance of difference, the Waller–Duncan test was used to determine the statistical differences between means, and *p* < 0.05 was considered statistically significant. Principal component analysis, cluster analysis, and regression analysis were handled by SPSS 23.0 software.

## 3. Results and Discussion

### 3.1. Physicochemical Traits of HB

The range, median, average, standard deviation (Std. deviation), and coefficient of variance (CV) of the physicochemical traits for the 76 HB are shown in Table S2. In general, each quality trait value of different HB varieties was widely distributed. For different indexes, the CV value ranged from 9.71–173.99%, indicating a large diversity among the tested sample.

There was a greater difference in amylose content among the tested varieties. The values ranged from 3.03% to 20.89%. (average: 15.81 ± 3.15%, CV: 19.09%). n particular, the amylose contents of CDU-57 and CDU-37 were far lower than the average level, with values of 3.03% and 4.37%, respectively. Compared with amylopectin, amylose has a relatively simple structure and stronger stability, which means amylose is not easily enzymatically hydrolyzed and digested [20]. Fat is an important energy storage component and supply substance in our body. Fat in food not only provides a sense of fullness, but also has a significant impact on the texture and sensory properties of food due to its emulsifying and water-holding properties [21]. Table 1 shows that the fat content among tested samples was relatively large, with a CV value of 44.28%. The CDU-47 and CDU-57 were 6.05% and 7.05%, respectively, which is much higher than the mean value (2.35 ± 1.04%). The protein content ranged from 3.99–8.99% (average: 6.59 ± 1.30%, CV: 19.76%). Protein plays an important role in food processing. It is related to many processing properties, such as solubility, foaming, gelling, and emulsifying. Protein also could affect the starch gelatinization and digestion through the interaction between the two [22]. The β-glucan is commonly considered as a characteristic substance of HB. In this study, the β-glucan content ranged from 3.33–8.97%, with an average of 4.57 ± 0.77%. The β-glucan content of CDU-37 (6.69%) and CDU-57 (8.97%) were significantly higher than others. The HB β-glucan has various biological activities [4,23], and its high viscosity and ability to prevent gluten formation through water absorption have a significant impact on processing [24]. In recent years, dietary fiber has attracted widespread attention from consumers because of its functions such as promoting intestinal peristalsis, enhancing satiety, and adjusting intestinal flora. Its ability to maintain food form and affect texture is also very significant in the field of food processing [25]. The average value of total dietary fiber content for tested samples was 20.50 ± 3.51%. The total phenolic average content was 336.06 ± 147.47 mg·100 g^−1^ and ranged from 62.26 mg·100 g^−1^ to 609.66 mg·100 g^−1^. In terms of the composition of phenolic, the free phenolics and bounded phenolics ranged from 28.19–385.58 mg·100 g^−1^ (average: 196.54 ± 92.83 mg·100 g^−1^) and 34.07–232.08 mg·100 g^−1^ (average: 139.51 ± 57.06 mg·100 g^−1^), respectively. The elements, including K, Mg, Ca, Fe, Na, Mn, Zn, Cu, Mo, Co, and Se, also were detected in this study (Table S2). Se has various functions, such as preventing various cancers [26], protecting the nervous system [27], enhancing immunity [28] and scavenging free radicals [29]. Referring to the selenium content standard (0.004–0.030 mg·100 g^−1^) by the National Standard of the People’s Republic of China-Rich selenium paddy [30], 36 out of 76 samples meet this standard, with a selenium rich rate of 39.47%.

### 3.2. Physicochemical Quality Traits of Different Colors HB

Previous studies have shown that HB with different seeds colors varied in terms of their physical and chemical traits [31]. Through cluster analysis based on the L*, a*, and b* of HB seeds, the tested samples were divided into five groups. In terms of visual colors, the five groups included the grey group (30 samples), yellow group (19 samples), brown group (14 samples), black group (12 samples), and red group (only 1 sample, not participating in subsequent analysis) (Table S3). Comparing the physicochemical traits of HB of different colors (Table 1), the contents of fat, free phenolics, bounded phenolics, and total phenolics increased significantly as the color deepened. The protein and total dietary fiber content showed a slight upward trend as the color changed from light to dark. For example, the average free phenolics and bounded phenolics ratio of the black group was 283.04 ± 40.81 mg·100 g^−1^ and 195.41 ± 23.82 mg·100 g^−1^, respectively, which was 2.6-fold and 2.3-folds greater compared to the grey group. The protein and total dietary fiber showed a less obvious upward trend, and the content of amylose decreased as the color deepened. For example, the average protein of the grey group was 5.75 ± 1.18%, which was significantly lower than that of the yellow, brown, and black groups, and there was no significant difference between the yellow, brown, and black groups. Total dietary fiber and amylose are similar to protein. The maximum average total dietary fiber was 23.61 ± 2.33% of the black group, and the maximum average amylose was 18.10 ± 2.20% of the grey group. However, there was no significant difference between various colors groups in terms of the moisture, total starch, amylopectin, and β-glucan.

The color of HB is mainly attributed to the pigments deposited in the pericarp and aleurone layer [32]. The light colored HB seeds mainly contain the colorless proanthocyanins [33]. The dark seeds are rich in anthocyanins in glumes and pericarp. Black seeds contain melanin in glumes and pericarp and blue seeds include anthocyanins in the aleurone layer [34].

In terms of elements, the Mg, K, Ca, and Zn content increased along with the color becoming darker. As shown in Table 1, the content of magnesium in the grey, yellow, brown, and black group was 92.60 ± 47.25 mg·100 g^−1^, 145.92 ± 38.85 mg·100 g^−1^, 142.61 ± 23.15 mg·100 g^−1^, and 115.51 ± 27.81 mg·100 g^−1^, respectively. Previous studies have shown that for accumulation of anthocyanins, conductively, Mg and anthocyanins will usually form the stable complex [35]. K and Ca not only played an important role in the accumulation of sugars [36], but also affected the production of anthocyanins. Hence, the darker color groups may possess higher levels of magnesium, potassium, and calcium. In addition, Fe, Co, Cu, Mo, and Se showed no obvious changing trend among the different colored groups.

### 3.3. Texture Parameters of Different Colors Tsampa

The three color parameters (L*, a*, and b*) and seven texture parameters (hardness, adhesiveness, springiness, cohesiveness, gumminess, chewiness, and resilience) of Tsampa were measured and the data are shown in Table S4. According to the color data, Tsampa is also divided into four groups, which is consistent with the seeds. The variances for each Tsampa texture’s parameters spanned a wide range among the samples, and the most significant contrasts were found in the adhesiveness and chewiness. The adhesiveness ranged from 0.67 g·s to 26.47 g·s; the chewiness ranged from 2.90 to 13.61. The variable amplitude of adhesiveness was the largest with a CV value of 55.56%, whereas the change in hardness was the smallest (CV: 13.56%). Among all samples, SS-17 was the stickiest, SS-02 was the hardest, and SS-24 had the strongest gumminess. CDU-57 had the highest springiness, cohesiveness, chewiness, and resilience.

**Figure 1:**
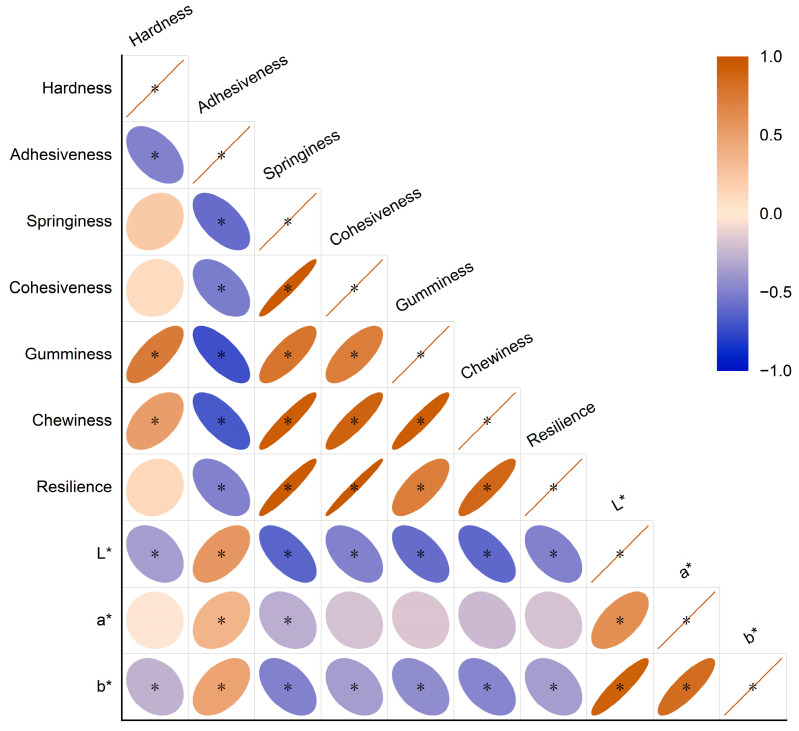
Correlation coefficient heat map of 10 quality indexes of Tsampa. Brown indicates positive correlation; blue indicates negative correlation; and the circle area indicates correlation coefficient; when the correlation coefficient is 1, the color is the deepest and the area is the smallest. (* *p* < 0.05).

By comparing the texture parameters of Tsampa of different colors, it was found that with color deepening, the hardness, springiness, cohesiveness, gumminess, chewiness, and resilience were increasing. However, the adhesiveness declined (Figure 1, Table S5). For example, the average hardness of the grey group was 3125.82 ± 421.41 g. It was significantly lower than the other three groups (yellow group: 3565.01 ± 400.32 g; light brown group: 3510.98 ± 514.16 g; dark brown group: 3570.24 ± 461.33 g) and there was no significant difference among the yellow, light brown, and dark brown groups. The average adhesiveness of the grey group was 14.61 ± 6.55 g·s, which was 1.8-fold greater compared to the dark brown group (Table 2).

### 3.4. Construction of Tsampa Comprehensive Quality Evaluation Model

Principal component analysis (PCA), as a commonly used data analysis method, can extract the main feature components of data and is commonly used for dimensionality reduction of high-dimensional data. PCA was used to reflect the quality of Tsampa based on 10 indicators (seven texture parameters and three color parameters). On the basis of the correlation and weighted coefficients (Table S6), a model for evaluating Tsampa’s comprehensive quality was developed. A linear combination coefficient was calculated for each principal component based on the load amounts and corresponding eigenvalues, and eigenvalues greater than 1 were used as screening conditions to obtain new principal components. As is shown in Table 3, the weighing and normalizing the principal component coefficients, and the evaluation model of the comprehensive quality of Tsampa was obtained in an accumulated manner. The equation for the evaluation model was $Y=-0.200x_{1}-0.097x_{2}-0.165x_{3}+0.140x_{4}-0.207x_{5}+0.249x_{6}+0.231x_{7}+0.251x_{8}+0.264x_{9}+0.231x_{10}.$ The vectors *x*_1_*–x*_10_ were defined as the following: L* (*x*_1_), a* (*x*_2_), b* (*x*_3_), hardness (*x*_4_), adhesiveness (*x*_5_), springiness (*x*_6_), cohesiveness (*x*_7_), gumminess (*x*_8_), chewiness (*x*_9_), resilience (*x*_10_). Y was regarded as the score of the comprehensive quality of Tsampa, and the higher the value of Y, the better the quality of the Tsampa. It can be seen from the model that hardness, springiness, cohesiveness, gumminess, chewiness, and resilience had positive effects on the comprehensive quality of Tsampa, while adhesiveness, L*, a*, and b* were opposite.

Based on the above model, all Tsampa were divided into three grades by cluster analysis (Table 4), Grade I, including 22 samples, represents good with Y > 0.8904; Grade II, including 23 samples, represents qualified with −0.8658 < Y ≤ 0.8904; Grade III;, including 25 samples, represents unqualified with Y ≤ −0.8658. Specific to texture parameters, samples in Grade I (good) showed low adhesiveness, appropriate hardness, good springiness, suitable cohesiveness, low gumminess, high chewiness, and resilience (Table S7). For example, CDU-49 was determined as a high-quality product with Y at 3.2639, hardness at 4217.7 g, adhesiveness at 3.33 g·s, springiness at 0.0553, cohesiveness at 0.0505, gumminess at 207.42 g, chewiness at 11.88 g, and resilience at 0.0168. Samples in Grade II (qualified), such as CDU-22 and CDU-06, had all parameters at the middle level. However, samples in Grade III were assessed as unqualified materials, due to their higher adhesion that causes teeth to stick easily. For example, the average value of adhesion for Grade III was 3.1-folds and 1.8-folds greater compared to Grade I and Grade II, respectively.

### 3.5. Construction of Tsampa Comprehensive Quality Prediction Model

The comprehensive quality of Tsampa and the physicochemical traits of HB (Table 5) were analyzed using correlation analysis. The comprehensive quality of Tsampa was positively correlated with the protein (r = 0.417, *p* < 0.01), Mg (r = 0.338, *p* < 0.01), a* (r = 0.322, *p* < 0.001), b* (r = 0.300, *p* < 0.005), fat (r = 0.300, *p* < 0.005), Zn (r = 0.294, *p* < 0.005), free phenolic (r = 0.288, *p* < 0.005), total phenolic (r = 0.285, *p* < 0.005), Mn (r = 0.272, *p* < 0.005), bound phenolic (r = 0.269, *p* < 0.005), and K (r = 0.265, *p* < 0.005). However, the amylose (r = −0.542, *p* < 0.01) and total starch (r = −0.251, *p* < 0.05) were negatively correlated with the comprehensive quality of Tsampa. The outcomes revealed that these 13 quality indicators could apply to predicting the comprehensive quality of Tsamba.

The backward regression analysis as a multiple linear regression method was used for selecting the key variables. Firstly, a model containing all independent variables was established. A critical *p*-value was used to verify the accuracy of the prediction by removing variables that did not have a significant impact on the model step-by-step after evaluation. In this study, 56 of the 70 Tsampa samples were randomly selected as the calibration for constructing the regression model. The remaining 14 (numbered V1–V14) were used as verification. We set the comprehensive quality of Tsampa (Y) as a dependent variable and the 25 physicochemical traits of HB as the independent variable to establish a predictive model by means of stepwise regression analysis.

Then, the following six HB key quality traits which affected the quality of Tsampa significantly were found: moisture, amylose, protein, β-glucan, a*, and b*. The prediction equation of the model was:

$$F=-4.047+0.307X_{1}-0.170X_{2}+0.391X_{3}+0.562X_{4}+0.322X_{5}-0.089X_{6}.$$

In this model, R^2^ = 0.681, *p* < 0.01, and we defined the vectors as the following: moisture, *X*_1_; amylose, *X*_2_; protein, *X*_3_; β-glucan, *X*_4_; a*, *X*_5_; and b*, *X*_6_ (Table S8).

Adhesiveness, springiness, gumminess, and chewiness had a critical impact on the quality of Tsampa with high-weight indicators in the comprehensive quality evaluation model. The correlation analysis results in Table 5 show that amylose was negatively correlated with springiness (r = −0.759, *p* < 0.01), cohesiveness (r = −0.758, *p* < 0.01), gumminess (r = −0.441, *p* < 0.01), chewiness (r = −0.617, *p* < 0.01), and resilience (r = −0.753, *p* < 0.01), and positively correlated with the adhesiveness of Tsampa (r = 0.274, *p* < 0.05). The higher amylose content leads to stronger water absorption, which can affect the formation of its network structure by competing with the protein for water [37]. Results showed that amylose had a negative impact on the comprehensive quality of Tsampa, which may be attributed to the water holding capacity reducing the compactness and enhancing the adhesiveness of Tsampa. The protein was significantly positively correlated with hardness (r = 0.326, *p* < 0.01), springiness (r = 463, *p* < 0.01), cohesiveness (r = 0.421, *p* < 0.01), gumminess (r = 0.542, *p* < 0.01), chewiness (r = 0.507, *p* < 0.01), and resilience (r = 0.392, *p* < 0.01), while negatively correlated with adhesiveness (r = −0.387, *p* < 0.01). The results indicated that the higher protein content results in a better internal texture for Tsampa. However, the protein of HB cannot form a gluten structure. It is supposed that protein affects viscosity, adhesiveness, and other properties to improve the cohesiveness texture. β-glucan was significantly positively correlated with springiness (r = 0.314, *p* < 0.01), cohesiveness (r = 0.410, *p* < 0.01), and resilience (r = 0.430, *p* < 0.01), while negatively correlated with hardness (r = −0.391, *p* < 0.01). As a characteristic substance of HB, β-glucan has a variety of biological activities [38]. The combined action of protein and β-glucan is beneficial for forming a harder, stronger, and more stable gluten network structure [39] which may be the key to positively affecting the quality of Tsampa. Hence, the optimal combination of indicators in the prediction model were representative and typical, which suggests that the products’ quality can be accurately predicted.

### 3.6. Verification of Tsampa Comprehensive Quality Predictive Model

**Figure 2:**
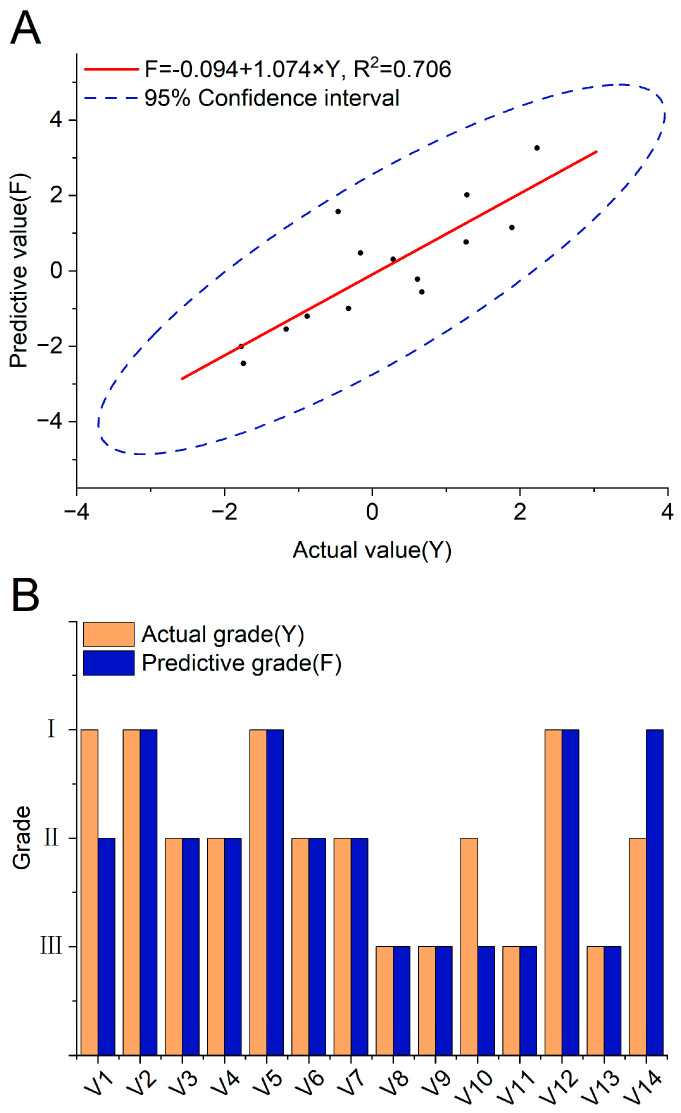
Verification of the Tsampa comprehensive quality predictive model. (**A**) The correlation analysis between predictive score F and actual score Y based on samples in verification. (**B**) The consistence of predictive and actual grade based on samples in verification.

The prediction scores F of Tsampa in verification were obtained through the prediction equation in Section 3.6 to verify the accuracy and stability of the Tsampa comprehensive quality predictive model. Based on the actual quality values Y of Tsampa obtained earlier, it was found that the predicted values F of the validation sample were close to the actual values Y, with R^2^ being 0.706. And, all points were within the 95% confidence interval (Figure 2A). The 14 verified varieties were classified based on standards of Y in Table 4. In total, 11 of 14 samples were successfully predicted, and the predictive accuracy was 78.57% (Figure 2B, Table S9). The results proved that the predictive model could accurately predict the comprehensive quality of Tsampa through the physicochemical traits of HB, and further confirmed that HB with a dark color and high β-glucan and protein content and low amylose content, such as CDU-57, CDU-49, CDU-37, SS-24, would be ideal raw material for processing high-quality Tsampa.

## 4. Conclusions

In this study, 25 indicators of 76 HB samples were systematically analyzed. Various physicochemical traits of raw material figuring prominently in Tsampa quality were certified. Correlation analysis showed that amylose, protein, and a* were the three physicochemical traits of highland barley which mostly related to the comprehensive quality of Tsampa. Then, a model based on the physicochemical traits of HB for predicting the comprehensive quality of Tsampa was constructed. The validation results indicated that the model was relatively accurate and stable. According to the analysis results, HB with a dark color and high β-glucan and protein content and low amylose content would be ideal raw material for high-quality Tsampa. Our study built the approach for the evaluation of HB-appropriate processing and may provide reference for processing specific HB.

## Figures and Tables

**Table 1:** Physicochemical traits of highland barley with different colors.

Index	Grey	Yellow	Brown	Black
L*	74.41 ± 8.63 ^a^	60.01 ± 5.41 ^b^	47.84 ± 4.5 ^c^	39.34 ± 2.13 ^d^
a*	0.91 ± 0.74 ^c^	4.38 ± 0.78 ^a^	4.34 ± 0.73 ^a^	3.42 ± 0.33 ^b^
b*	28.39 ± 2.97 ^b^	37.12 ± 3.77 ^a^	27.23 ± 3.28 ^b^	16.64 ± 1.98 ^c^
Moisture (%)	11.21 ± 1.52	10.31 ± 0.75	10.41 ± 0.46	10.98 ± 0.92
Fat (%)	2.10 ± 0.67 ^b^	2.38 ± 1.42 ^a,b^	2.22 ± 0.64 ^a,b^	3.04 ± 1.30 ^a^
Total starch (%)	52.66 ± 4.90	50.45 ± 4.96	48.54 ± 5.00	52.38 ± 4.32
Amylose (%)	18.10 ± 2.20 ^a^	13.78 ± 3.85 ^b^	13.70 ± 1.45 ^b^	15.81 ± 1.01 ^b^
Amylopectin (%)	34.56 ± 4.79	36.66 ± 5.67	34.84 ± 4.48	36.56 ± 3.97
Protein (%)	5.75 ± 1.18 ^c^	7.18 ± 1.02 ^a,b^	7.64 ± 0.72 ^a^	6.66 ± 1.2 ^a,b^
β-Glucan (%)	4.65 ± 0.44	4.77 ± 1.23	4.14 ± 0.56	4.50 ± 0.53
Total dietary fiber (%)	19.29 ± 4.40 ^b^	20.48 ± 1.85 ^b^	20.24 ± 2.41 ^b^	23.61 ± 2.33 ^a^
Free phenolic (mg·100 g^−1^)	107.08 ± 46.62 ^c^	231.14 ± 68.51 ^b^	254.94 ± 62.56 ^a,b^	283.04 ± 40.81 ^a^
Bound phenolic (mg·100 g^−1^)	82.33 ± 32.14 ^c^	158.28 ± 38.29 ^b^	183.80 ± 23.67 ^a,b^	195.41 ± 23.82 ^a^
Total phenolic (mg·100 g^−1^)	189.42 ± 77.44 c	389.42 ± 102.92 ^b^	438.74 ± 80.01 ^a,b^	478.45 ± 57.18 ^a^
Na (mg·100 g^−1^)	31.11 ± 23.51	31.92 ± 18.84	21.11 ± 14.54	27.35 ± 14.19
Mg (mg·100 g^−1^)	92.60 ± 47.25 ^b^	145.92 ± 38.85 ^a^	142.61 ± 23.15 ^a^	115.51 ± 27.81 ^a,b^
K (mg·100 g^−1^)	407.72 ± 142.79 ^b^	637.72 ± 159.68 ^a^	658.81 ± 115.46 ^a^	574.42 ± 154.49 ^a^
Ca (mg·100 g^−1^)	47.50 ± 21.15 ^b^	65.61 ± 20.55 ^a^	60.52 ± 14.71 ^a,b^	58.31 ± 17.55 ^a,b^
Mn (mg·100 g^−1^)	2.57 ± 1.26	2.89 ± 0.83	2.40 ± 0.54	2.64 ± 1.15
Fe (mg·100 g^−1^)	76.03 ± 95.69 ^a^	26.34 ± 54.68 ^a,b^	10.82 ± 8.88 ^b^	21.96 ± 46.49 ^a,b^
Co (mg·100 g^−1^)	0.1828 ± 0.1737 ^a^	0.0661 ± 0.1496 ^a,b^	0.0058 ± 0.0034 ^b^	0.0567 ± 0.1435 ^a,b^
Cu (mg·100 g^−1^)	1.18 ± 0.93 ^a^	0.86 ± 0.58 ^a,b^	0.98 ± 0.42 ^a,b^	0.39 ± 0.46 ^b^
Zn (mg·100 g^−1^)	2.87 ± 1.03 ^b^	3.74 ± 0.87 ^a,b^	4.01 ± 0.70 ^a^	3.24 ± 0.75 ^a,b^
Se (mg·100 g^−1^)	0.0026 ± 0.0036	0.0058 ± 0.0124	0.0049 ± 0.0052	0.0087 ± 0.0065
Mo (mg·100 g^−1^)	0.40 ± 0.44 ^a^	0.19 ± 0.44 ^a,b^	0.06 ± 0.05 ^b^	0.16 ± 0.24 ^a,b^

Different letters in the same line express statistically significant differences (*p* < 0.05).

**Table 2:** Texture parameters of Tsampa with different colors.

Index	Grey	Yellow	Light Brown	Dark Brown
Hardness (g)	3125.82 ± 421.41 ^b^	3565.01 ± 400.32 ^a^	3510.98 ± 514.16 ^a^	3570.24 ± 416.33 ^a^
Adhesiveness (g·s)	14.61 ± 6.55 ^a^	14.51 ± 6.02 ^a^	9.87 ± 5.21 ^a,b^	8.16 ± 6.32 ^b^
Springiness	0.0373 ± 0.0037 ^c^	0.0421 ± 0.0039 ^b,c^	0.0488 ± 0.0087 ^a^	0.0465 ± 0.0046 ^a,b^
Cohesiveness	0.0369 ± 0.004 ^c^	0.0411 ± 0.0044 ^b,c^	0.0475 ± 0.011 ^a^	0.0442 ± 0.0043 ^a,b^
Gumminess (g)	116.34 ± 26.11 ^b^	147.39 ± 27.86 ^a^	163.7 ± 25.20 ^a^	157.62 ± 29.34 ^a^
Chewiness (g)	4.43 ± 1.48 ^c^	6.29 ± 1.69 ^b^	8.04 ± 2.42 ^a^	7.44 ± 2.04 ^a,b^
Resilience	0.0123 ± 0.0011 ^c^	0.0138 ± 0.0013 ^b,c^	0.0159 ± 0.0037 ^a,b^	0.0147 ± 0.0014 ^a^

Different letters in the same line express statistically significant differences (*p* < 0.05).

**Table 3:** Weight distribution of Tsampa quality indexes and the comprehensive quality evaluation model.

	Tsampa Quality Index	PC_1_	PC_2_	PC_3_
Tsampa quality index weight coefficient	*x*_1_	−0.319	0.061	0.000
	*x*_2_	−0.182	0.102	0.028
	*x*_3_	−0.278	0.090	0.010
	*x*_4_	0.187	0.001	0.110
	*x*_5_	−0.306	0.008	−0.038
	*x*_6_	0.374	0.030	−0.033
	*x*_7_	0.346	0.045	−0.047
	*x*_8_	0.361	0.028	0.046
	*x*_9_	0.387	0.033	0.007
	*x*_10_	0.345	0.045	−0.045
PC weight coefficient		0.662	0.193	0.145
Evaluation model		Y = −0.200*x*_1_ − 0.097*x*_2_ − 0.165*x*_3_ + 0.140*x*_4_ − 0.207*x*_5_ + 0.249*x*_6_ + 0.231*x*_7_ + 0.251*x*_8_ + 0.264*x*_9_ + 0.231*x*_10_		

PC represents the principal component. The vectors *x*_1_*–x*_10_ were defined as the following: L* (*x*_1_), a* (*x*_2_), b* (*x*_3_), hardness (*x*_4_), adhesiveness (*x*_5_), springiness (*x*_6_), cohesiveness (*x*_7_), gumminess (*x*_8_), chewiness (*x*_9_), resilience (*x*_10_).

**Table 4:** The quality grade of Tsampa according to comprehensive score Y.

Grade	Quantity	Range	Number
Grade I	22	0.8904 < Y	CDU-17, CDU-18, CDU-19, CDU-34, CDU-37, CDU-38, CDU-47, CDU-49, CDU-50, CDU-51, CDU-52, CDU-53, CDU-54, CDU-56, CDU-57, CDU-58, SS-11, SS-20, SS-23, SS-24, SS-29, SS-32
Grade II	23	−0.8658 < Y ≤ 0.8904	CDU-15, CDU-16, CDU-20, CDU-22, CDU-35, CDU-36, CDU-41, CDU-42, CDU-43, CDU-45, CDU-48, CDU-59, CDU-60, SS-02, SS-03, SS-05, SS-06, SS-09, SS-10, SS-12, SS-14, SS-28, SS-35
Grade III	25	Y ≥ −0.8658	CDU-21, CDU-33, CDU-40, CDU-55, SS-01, SS-04, SS-07, SS-08, SS-13, SS-15, SS-16, SS-17, SS-18, SS-19, SS-21, SS-22, SS-25, SS-26, SS-27, SS-30, SS-31, SS-33, SS-34, SS-36, SS-37

**Table 5:** Correlation analysis of the texture quality of Tsampa and the physicochemical indexes of highland barley.

Index	Hardness	Adhesiveness	Springiness	Cohesiveness	Gumminess	Chewiness	Resilience	L* (Tsampa.)	a* (Tsampa.)	b* (Tsampa.)	Y
Moisture	0.321 ^b^	−0.178	0.043	0.060	0.296 ^a^	0.192	−0.007	0.106	0.118	0.092	0.222
Total starch	−0.072	−0.028	−0.200	−0.197	−0.175	−0.156	−0.231	0.124	−0.022	0.080	−0.251 ^a^
Amylose	0.071	0.274 ^a^	−0.759 ^b^	−0.758 ^b^	−0.441 ^b^	−0.617 ^b^	−0.753 ^b^	0.555 ^b^	0.165	0.387 ^b^	−0.542 ^b^
Amylopectin	−0.120	−0.209	0.297 ^a^	0.300 ^a^	0.112	0.248 ^a^	0.261 ^a^	−0.240 ^a^	−0.131	−0.174	0.101
Fat	0.059	−0.073	0.372 ^b^	0.367 ^b^	0.257 ^a^	0.348 ^b^	0.406 ^b^	−0.328 ^b^	−0.225	−0.311 ^b^	0.300 ^a^
Protein	0.326 ^b^	−0.387 ^b^	0.463 ^b^	0.421 ^b^	0.542 ^b^	0.507 ^b^	0.392 ^b^	−0.472 ^b^	−0.140	−0.346 ^b^	0.417 ^b^
β-Glucan	−0.391 ^b^	0.132	0.314 ^b^	0.410 ^b^	−0.064	0.167	0.430 ^b^	0.127	0.038	0.135	0.213
Total dietary fibre	0.188	−0.185	0.210	0.110	0.158	0.206	0.103	−0.323 ^b^	−0.339 ^b^	−0.383 ^b^	0.075
Free phenol	0.272 ^a^	−0.421 ^b^	0.500 ^b^	0.401 ^b^	0.445 ^b^	0.491 ^b^	0.415 ^b^	−0.742 ^b^	−0.390 ^b^	−0.651 ^b^	0.288 ^a^
Bound phenol	0.253 ^a^	−0.462 ^b^	0.526 ^b^	0.423 ^b^	0.454 ^b^	0.509 ^b^	0.438 ^b^	−0.784 ^b^	−0.435 ^b^	−0.693 ^b^	0.269 ^a^
Total phenol	0.269 ^a^	−0.443 ^b^	0.517 ^b^	0.415 ^b^	0.455 ^b^	0.505 ^b^	0.430 ^b^	−0.770 ^b^	−0.413 ^b^	−0.677 ^b^	0.285 ^a^
Na	0.016	0.250 ^a^	−0.078	−0.071	−0.061	−0.069	−0.078	0.113	0.225	0.159	0.112
Mg	0.127	−0.198	0.404 ^b^	0.347 ^b^	0.303 ^a^	0.335 ^b^	0.329 ^b^	−0.338 ^b^	−0.047	−0.232	0.338 b
K	0.211	−0.276 ^a^	0.363 ^b^	0.271 ^a^	0.306 ^b^	0.320 ^b^	0.262 ^a^	−0.443 ^b^	−0.103	−0.305 ^a^	0.265 ^a^
Ca	0.151	−0.118	0.235 ^a^	0.156	0.181	0.203	0.144	−0.254 ^a^	−0.049	−0.175	0.203
Mn	0.219	0.233	0.001	−0.021	0.098	0.046	−0.008	−0.008	0.213	0.080	0.272 ^a^
Fe	0.068	0.501 ^b^	−0.226	−0.175	−0.122	−0.172	−0.155	0.351 ^b^	0.302 ^a^	0.339 ^b^	0.148
Co	0.047	0.484 ^b^	−0.246 ^a^	−0.185	−0.123	−0.179	−0.165	0.360 ^b^	0.353 ^b^	0.360 ^b^	0.136
Cu	−0.063	0.393 ^b^	−0.263 ^a^	−0.195	−0.214	−0.225	−0.200	0.337 ^b^	0.332 ^b^	0.366 ^b^	0.007
Zn	0.159	−0.139	0.285 ^a^	0.208	0.237 ^a^	0.264 ^a^	0.212	−0.263 ^a^	0.084	−0.117	0.294 ^a^
Se	0.216	−0.181	0.073	0.042	0.183	0.154	0.071	−0.299 ^a^	−0.174	−0.231	0.034
Mo	0.000	0.528 ^b^	−0.258 ^a^	−0.203	−0.187	−0.215	−0.185	0.335 ^b^	0.285 ^a^	0.320 ^b^	0.086
L* (HB seeds)	−0.339 ^b^	0.523 ^b^	−0.482 ^b^	−0.346 ^b^	−0.471 ^b^	−0.480 ^b^	−0.344 ^b^	0.866 ^b^	0.581 ^b^	0.845 ^b^	−0.189
a* (HB seeds)	0.167	−0.370 ^b^	0.507 ^b^	0.438 ^b^	0.397 ^b^	0.455 ^b^	0.438 ^b^	−0.664 ^b^	−0.212	−0.490 ^b^	0.322 ^b^
b* (HB seeds)	−0.239 ^a^	0.209	0.115	0.210	−0.041	0.036	0.187	0.296 ^a^	0.600 ^b^	0.533 ^b^	0.300 ^a^

^a^ Represent the significance levels at *p* < 0.05. ^b^ Represent the significance levels at *p* < 0.01.

## Data Availability

The original contributions presented in the study are included in the article or supplementary material, further inquiries can be directed to the corresponding author.

## Supplementary Materials

The following supporting information can be downloaded at: https://www.mdpi.com/article/10.3390/foods13040613/s1.
