# Supplementary material for: The Quality Evaluation of Highland Barley and Its Suitability for Chinese Traditional Tsampa Processing
Source: Foods. 2024 Feb 18;13(4):613. doi: 10.3390/foods13040613 (PMC10887829; doi:10.3390/foods13040613)
Supplement: Supplementary file 1 [file foods-13-00613-s001.zip › Supplementary data.pdf]

Table S1 Basic information of tested HB.

Number	OS	Notes	Number	OS	Notes	Number	OS	Notes
CDU-14	Sichuan	RA	CDU-48	Qinghai	CS	SS-13	Tibet	VS
CDU-15	Tibet	CS	CDU-49	Qinghai	CS	SS-14	-	CS
CDU-16	Sichuan	VS	CDU-50	Qinghai	CS	SS-15	Tibet	CS
CDU-17	Tibet	VS	CDU-51	Qinghai	CS	SS-16	Tibet	VS
CDU-18	Tibet	CS	CDU-52	Qinghai	CS	SS-17	Qinghai	CS
CDU-19	Tibet	CS	CDU-53	Qinghai	CS	SS-18	Gansu	CS
CDU-20	Sichuan	VS	CDU-54	Qinghai	CS	SS-19	Qinghai	CS
CDU-21	Sichuan	CS	CDU-55	Qinghai	CS	SS-20	Tibet	CS
CDU-22	Sichuan	CS	CDU-56	Tibet	CS	SS-21	Tibet	CS
CDU-23	Sichuan	RA	CDU-57	Tibet	CS	SS-22	Tibet	CS
CDU-27	Sichuan	RA	CDU-58	Tibet	CS	SS-23	Tibet	CS
CDU-28	Sichuan	RA	CDU-59	Tibet	CS	SS-24	Tibet	CS
CDU-29	Sichuan	RA	CDU-60	Tibet	CS	SS-25	-	VS
CDU-33	Qinghai	CS	SS-01	Tibet	CS	SS-26	Shaanxi	VS
CDU-34	Sichuan	CS	SS-02	Qinghai	VS	SS-27	Tibet	CS
CDU-35	Sichuan	CS	SS-03	Qinghai	CS	SS-28	Qinghai	CS
CDU-36	Qinghai	VS	SS-04	Tibet	CS	SS-29	Tibet	CS
CDU-37	Sichuan	VS	SS-05	Gansu	CS	SS-30	Tibet	VS
CDU-38	Sichuan	CS	SS-06	Qinghai	VS	SS-31	Tibet	CS
CDU-39	Sichuan	RA	SS-07	Tibet	CS	SS-32	-	VS
CDU-40	Qinghai	CS	SS-08	Qinghai	CS	SS-33	-	CS
CDU-41	Qinghai	CS	SS-09	Qinghai	CS	SS-34	Qinghai	CS
CDU-42	Qinghai	CS	SS-10	Tibet	CS	SS-35	-	CS
CDU-43	Qinghai	CS	SS-11	Tibet	CS	SS-36	Tibet	CS
CDU-45	Tibet	CS	SS-12	Tibet	CS	SS-37	Tibet	CS
CDU-47	Qinghai	CS						

- Represents unknown locality. OS represents origin source. RA represents rare amount, HB with RA

marked were not made into Tsampa for subsequent analysis. CS represents calibration set. VS represents verification set.

Table S2 Physicochemical traits of 76 varieties highland barley.

Index	No.	Range	Median	Average	Std. deviation	CV (%)
L*	76	35.10-85.56	59.61	60.14	14.80	24.61
a*	76	-0.71-10.49	3.28	2.93	1.95	66.55
b*	76	13.54-46.78	29.39	28.52	7.14	25.04
Moisture (%)	76	9.07-14.70	10.47	10.77	1.18	10.96
Fat (%)	76	0.99-7.50	2.12	2.35	1.04	44.26
Total starch (%)	76	39.45-61.84	50.55	51.30	4.98	9.71
Amylose (%)	76	3.03-20.98	15.73	15.81	3.15	19.92
Amylopectin (%)	76	24.76-48.98	35.80	35.49	4.83	13.61
Protein (%)	76	3.99-8.99	6.50	6.59	1.30	19.73
β-glucan (%)	76	3.33-8.97	4.53	4.57	0.77	16.85
Total dietary fiber (%)	76	13.56-32.15	19.74	20.50	3.51	17.12
Free phenolic ($mg·100g^{-1}$)	76	28.19-385.58	208.60	196.543	92.83	47.23
Bound phenolic ($mg·100g^{-1}$)	76	34.07-232.08	148.28	139.51	57.06	40.90
Total phenolic ($mg·100g^{-1}$)	76	62.26-609.66	348.54	336.06	147.47	43.88
Na ($mg·100g^{-1}$)	76	2.86-108.05	25.06	29.00	19.57	67.48
Mg ($mg·100g^{-1}$)	76	35.89-270.25	112.17	118.89	44.76	37.65
K ($mg·100g^{-1}$)	76	293.14-1012.98	523.65	539	179.05	33.18
Ca ($mg·100g^{-1}$)	76	20.49-127.28	53.70	56.17	20.35	36.23
Mn ($mg·100g^{-1}$)	76	0.90-6.07	2.39	2.62	1.03	39.31
Fe ($mg·100g^{-1}$)	76	0.00-321.66	7.73	42.14	73.32	173.99
Co ($mg·100g^{-1}$)	76	0.00-0.5748	0.0049	0.0987	0.1587	160.74
Cu ($mg·100g^{-1}$)	76	0.00-4.90	0.75	0.92	0.75	81.52
Zn ($mg·100g^{-1}$)	76	1.37-5.19	3.26	3.36	0.99	29.46

Se (mg·100g^{-1})	76	0.00-0.0544	0.0025	0.0048	0.0076	160.02
Mo (mg·100g^{-1})	76	0.01-1.96	0.07	0.24	0.39	162.50

Table S3 Color classification of HB seeds.

Group	Quantity	Number
Gary	30	SS-01、SS-02、SS-03、SS-04、SS-05、SS-06、SS-07、SS-08、SS-09、SS-10、SS-11、SS-13、SS-15、SS-16、SS-18、SS-19、SS-20、SS-21、SS-22、SS-23、SS-25、SS-26、SS-27、SS-28、SS-30、SS-31、SS-34、SS-35、SS-36、SS-37
Yellow	19	CDU-19、CDU-20、CDU-21、CDU-22、CDU-33、CDU-34、CDU-35、CDU-36、CDU-37、CDU-40、CDU-41、CDU-56、CDU-57、CDU-59、CDU-60、SS-14、SS-17、SS-32、SS-33
Brown	14	CDU-14、CDU-15、CDU-16、CDU-17、CDU-18、CDU-23、CDU-27、CDU-28、CDU-29、CDU-39、CDU-42、CDU-43、CDU-51、SS-12
Black	12	CDU-38、CDU-45、CDU-47、CDU-48、CDU-49、CDU-50、CDU-52、CDU-53、CDU-54、CDU-55、SS-24、SS-29
Red	1	CDU-58

Table S4 Colors and texture characteristics of Tsampa.

Index	No.	Range	Median	Average	Std. deviation	CV (%)
L*	70	39.56 - 59.17	48.59	48.51	5.34	11.01
a*	70	2.21 - 7.32	4.75	4.65	1.30	27.96
b*	70	1.77 - 13.74	9.35	8.36	3.39	40.55
Hardness (g)	70	2308.78 - 4440.89	3514.70	3471.67	470.77	13.56
Adhesiveness (g·sec)	70	0.67 - 26.49	11.21	11.77	6.54	55.56
Springiness	70	0.0320 - 0.0735	0.0439	0.0440	0.0069	15.68
Cohesiveness	70	0.0333 - 0.0790	0.0419	0.0427	0.0074	17.33
Gumminess (g)	70	86.56 - 212.43	147.36	148.22	31.43	21.20
Chewiness (g)	70	2.90 - 13.61	6.48	6.67	2.29	34.33
Resilience	70	0.0110 - 0.0270	0.0140	0.0143	0.0024	16.78

Table S5 Color classification of Tsampa.

Group	Quantity	Number
G	13	SS-01、SS-03、SS-06、SS-07、SS-08、SS-16、SS-21、SS-25、SS-27、SS-30、SS-34、SS-36、SS-37
Y	22	CDU-20、CDU-21、CDU-33、CDU-35、CDU-59、CDU-60、SS-02、SS-09、SS-10、SS-11、SS-13、SS-15、SS-17、SS-18、SS-19、SS-20、SS-22、SS-23、SS-26、SS-28、SS-33、SS-35
LB	17	CDU-15、CDU-16、CDU-19、CDU-22、CDU-34、CDU-37、CDU-40、CDU-41、CDU-42、CDU-56、CDU-57、CDU-58、SS-05、SS-12、SS-14、SS-31、SS-32
DB	18	CDU-17、CDU-18、CDU-36、CDU-38、CDU-43、CDU-45、CDU-47、CDU-48、CDU-49、CDU-50、CDU-51、CDU-52、CDU-53、CDU-54、CDU-55、SS-04、SS-24、SS-29

Table S6 Correlation coefficient of quality indexes of Tsampa.

	Hardness	Adhesiveness	Springiness	Cohesiveness	Gumminess	Chewiness	Resilience	L*	a*	b*
Hardness	1	-0.499[b]	0.216	0.086	0.738[b]	0.506[b]	0.114	-0.360[b]	-0.026	-0.259[a]
Adhesiveness	-0.499[b]	1	-0.594[b]	-0.507[b]	-0.714[b]	-0.692[b]	-0.501[b]	0.545[b]	0.355[b]	0.478[b]
Springiness	0.216	-0.594[b]	1	0.961[b]	0.780[b]	0.935[b]	0.946[b]	-0.622[b]	-0.284[a]	-0.493[b]
Cohesiveness	0.086	-0.507[b]	0.961[b]	1	0.715[b]	0.887[b]	0.980[b]	-0.502[b]	-0.187	-0.365[b]
Gumminess	0.738[b]	-0.714[b]	0.780[b]	0.715[b]	1	0.936[b]	0.715[b]	-0.589[b]	-0.162	-0.435[b]
Chewiness	0.506[b]	-0.692[b]	0.935[b]	0.887[b]	0.936[b]	1	0.880[b]	-0.618[b]	-0.231	-0.469[b]
Resilience	0.114	-0.501[b]	0.946[b]	0.980[b]	0.715[b]	0.880[b]	1	-0.502[b]	-0.188	-0.370[b]
L*	-0.360[b]	0.545[b]	-0.622[b]	-0.502[b]	-0.589[b]	-0.618[b]	-0.502[b]	1	0.610[b]	0.912[b]
a*	-0.026	0.355[b]	-0.284[a]	-0.187	-0.162	-0.231	-0.188	0.610[b]	1	0.846[b]
b*	-0.259[a]	0.478[b]	-0.493[b]	-0.365[b]	-0.435[b]	-0.469[b]	-0.370[b]	0.912[b]	0.846[b]	1

[a] Represent the significance levels at $p<0.05$.

[b] Represent the significance levels at $p<0.01$.

Table 7 Differences in color and texture characteristics of three grades of Tsampa.

Index	Grade I	Grade II	Grade III
L*	43.62 ± 2.97[c]	48.11 ± 4.00[b]	53.19 ± 3.83[a]
a*	3.79 ± 1.14[b]	4.91 ± 1.42[a]	5.17 ± 0.93[a]
b*	5.53 ± 2.57[c]	8.34 ± 3.05[b]	10.86 ± 2.21[a]
Hardness (g)	3746.56 ± 516.14[a]	3533.25 ± 347.17[b]	3173.1 ± 357.52[c]
Adhesiveness (g·sec)	5.78 ± 3.16[c]	10.52 ± 4.29[b]	18.2 ± 4.48[a]
Springiness	0.05 ± 0.01[a]	0.04 ± 0.00[b]	0.04 ± 0.00[c]
Cohesiveness	0.05 ± 0.01[a]	0.04 ± 0.00[b]	0.04 ± 0.00[c]
Gumminess (g)	180.94 ± 17.72[a]	151.38 ± 15.03[b]	116.51 ± 17.89[c]
Chewiness (g)	9.21 ± 1.67[a]	6.67 ± 0.76[b]	4.42 ± 0.96[c]
Resilience	0.02 ± 0.00[a]	0.01 ± 0.00[b]	0.01 ± 0.00[c]

Table S8 Partial regression coefficients of key indicators.

Parameter	Partial regression coefficient	Std	Standard regression coefficient	t value	p value	Tolerance	VIF
constant	-4.047	2.941		-1.376	0.175		
Moisture	0.307	0.148	0.181	2.072	0.043	0.720	1.389
Amylose	-0.170	0.066	-0.311	-2.581	0.013	0.447	2.235
Protein	0.391	0.148	0.305	2.639	0.011	0.488	2.050
β-Glucan	0.562	0.209	0.266	2.685	0.010	0.664	1.507
a*	0.322	0.091	0.377	3.519	0.001	0.568	1.760
b*	-0.089	0.019	-0.406	-4.598	0.000	0.835	1.198

Table S9 Analysis on the prediction results of Tsampa in validation set.

	Actual value (Y)	Actual grade	Prediction value (F)	Prediction grade	Contrast
V1	1.272	I	0.764	II	Inconsistent
V2	1.283	I	2.015	I	Consistent
V3	0.673	II	-0.556	II	Consistent
V4	0.614	II	-0.221	II	Consistent
V5	2.232	I	3.256	I	Consistent
V6	0.285	II	0.308	II	Consistent
V7	-0.159	II	0.475	II	Consistent
V8	-1.772	III	-2.011	III	Consistent
V9	-1.743	III	-2.450	III	Consistent
V10	-0.322	II	-0.997	III	Inconsistent
V11	-0.882	III	-1.200	III	Consistent
V12	1.890	I	1.149	I	Consistent
V13	-1.166	III	-1.545	III	Consistent
V14	-0.460	II	1.569	I	Inconsistent
Accuracy			78.57%		
